# Clinical validation of controlled exposure to cat dander in the Specialized Particulate Control Environmental Exposure Unit (SPaC-EEU)

**DOI:** 10.1186/s13223-025-00978-z

**Published:** 2025-08-06

**Authors:** Lubnaa Hossenbaccus, Sophia Linton, Sarah Garvey, Hannah Botting, Terry Walker, Lisa Steacy, Anne K. Ellis

**Affiliations:** 1 Department of Medicine, Queen’s University, Kingston, ON Canada; 2 Allergy Research Unit, Kingston Health Sciences Centre - KGH Site,, Kingston, ON Canada; 3 Department of Biomedical and Molecular Sciences, Queen’s University, Kingston, ON Canada

**Keywords:** Cat allergen-induced allergic rhinitis, Specialized Particulate Control Environmental Exposure Unit, Clinical validation, Controlled allergen challenge facility, Fel d 1, Cat dander, Allergy

## Abstract

**Background:**

Cat allergen is the second most common cause of perennial allergic rhinitis. Despite its prevalence (~ 20% of the population), many patients continue to suffer from persistent symptoms due to constant exposure to cat allergens that reduce treatment efficacy. Modelling of the disease can improve our understanding of its onset and progression. The Specialized Particulate Control Environmental Exposure Unit (SPaC-EEU) is a controlled allergen challenge facility that has recently undergone a successful technical validation for cat dander exposure, measuring *Felis domesticus* 1 (Fel d 1). We then sought to perform a clinical validation with cat-allergic and non-allergic participants.

**Methods:**

This study consisted of 3 visits. Recruited participants attended a Screening visit where eligibility was assessed, and a skin prick test (SPT) was completed. Successfully screened cat-allergic and non-allergic participants were invited back for the Allergen Exposure visit. They attended one of two 3-hour cat dander exposure Sessions in the SPaC-EEU, due to space limitations, with a target Fel d 1 concentration of 70 ng/m^3^. Fel d 1 concentrations were collected using air sampling cassettes and processed using a Fel d 1-specific ELISA. Real-time particle counts were monitored using a laser particle counter (LPC). Participants recorded symptom scores at time points from baseline up to 24 h post-onset of allergen exposure. Participants returned to the research site for a 24-hour Follow-up visit. Allergic participants completed a cat exposure and Quality of Life questionnaire.

**Results:**

Forty-six (31 cat-allergic and 15 non-allergic) participants completed this study. Allergic participants had significantly larger (*p* < 0.0001) SPT wheals to cat hair than non-allergic controls. Twenty-five participants attended the first Session (mean Fel d 1 = 35.7 ng/m^3^), and 21 participants attended the second Session (mean Fel d 1 = 102.3 ng/m^3^). No significant differences in symptom and safety scores were observed between Sessions, hence participants’ data were pooled. Allergic participants experienced significantly elevated (*p* < 0.05) Total Nasal Symptom Scores and Total Rhinoconjunctivitis Symptom Scores from 15 min to 24-h post-onset of allergen exposure and significantly decreased (*p* < 0.05) percent change in Peak Nasal Inspiratory Flow from 1 to 3 hours, compared to non-allergic controls. Mean Quality of Life scores were different between phenotypes, unimpacted by whether or not one lived with a cat.

**Conclusion:**

The SPaC-EEU can safely produce clinically relevant nasal symptoms in only cat-allergic participants, highlighting its use for modelling cat allergen-induced allergic rhinitis.

**Supplementary Information:**

The online version contains supplementary material available at 10.1186/s13223-025-00978-z.

## Background

Allergic rhinitis (AR), colloquially known as “hayfever” or simply “allergies”, is a disease whereby exposure to allergen results in inflammation of the nasal and ocular mucosae following immunologic sensitization [1]. Cat allergens, classified as *Felis domesticus* (Fel d) allergens 1 through 8, are the second most common cause of perennial sensitization, estimated to impact between 10 and 30% of the population [2]. The most prevalent allergen is Fel d 1, a glycoprotein secreted through the salivary, anal, and sebaceous glands of cats and spread onto fur and hair through grooming behaviours. Cat allergens tend to travel on small particles, usually between 5 and 10 microns in size, and due to their static-prone nature, they cling to clothing and disperse within the environment [3, 4]. This leads to the presence of cat allergens in spaces without cats; even in homes without the presence of a cat, cat allergens have been detected at levels sufficient to trigger a response in allergic patients [5]. Fel d 1 levels decrease with the removal of a cat from a home, however, it takes at least 20 weeks to reach levels similar to homes without cats [6]. The constant exposure to cat allergens makes cat allergen-induced AR (cat-AR) more difficult to treat, as therapeutic efficacy is reduced, leaving many patients with continued symptoms despite medication use [7]. As a result, a better understanding of the immune response in cat allergies is needed and can be achieved through models of the disease.

Historically, “naturalistic” cat rooms have been used to model cat-AR. Participants are put in rooms where cats have lived or are present to observe the development of allergic symptoms. Such experimental setups have contributed to our knowledge of the pathophysiology of cat-AR as well as pharmacologic and non-pharmacologic interventions [8–10]. However, the lack of standardization and control of allergen concentrations of cat rooms necessitates a more robust model, such as an allergen exposure chamber (AEC) [11]. AECs are clinical facilities that distribute regulated concentrations of allergens in an enclosed space with a controlled environment. They simulate natural exposure while controlling for extraneous variables such as temperature and humidity. AECs can be leveraged to assess therapeutic efficacy and safety [12–15] allowing them to be important tools for the evaluation of new pharmacotherapies and allergen immunotherapies for cat-AR. Each AEC is uniquely designed and set up so independent validations are necessary for each facility [12, 16–18].

The Specialized Particulate Control Environmental Exposure Unit (SPaC-EEU), formerly referred to as the house dust mite EEU (HDM-EEU), is one of two AECs located in Kingston, Ontario Canada. With an anteroom to buffer the pressure differentials between the challenge room and surrounding spaces and prevent unwanted dissemination of allergen outside the AEC, the SPaC-EEU is designed for exposure to perennial allergens. It has successfully been technically and clinically validated for HDM exposure [19]. Following a successful technical validation to cat allergen in 2023, confirming the ability of the equipment to reproducibility distribute cat allergen in the facility [20]we hypothesized that the SPaC-EEU would be a suitable model of cat-AR through the elucidation of symptoms in allergic participants.

## Methods

The protocol for this study was reviewed and ethical approval was provided by the Queen’s University Health Sciences and Affiliated Teaching Hospitals Research Ethics Board. Study participants provided written, informed consent prior to any study-related procedures. Adolescent participants reviewed an assent form with their legal guardian reviewing the informed consent form.

### Participants

#### All participants

Participants on file in the Kingston Allergy Research database were approached to participate in this study. All participants were required to be between the ages of 12 to 65 years of age, able to understand and willing to provide written informed consent or assent, and able and willing to comply with study requirements. Participants of childbearing potential (Supplementary Data 1) were required to produce a negative urine pregnancy test at the Screening and Allergen Exposure visits. Participants matching any of the exclusionary criteria (Supplementary Data 2 and 3) were not included in the study.

#### Allergic participants

To be enrolled into the cat-allergic participant group, participants had to have a minimum 2-year self-reported history of AR symptoms to cats, with a positive skin prick test (SPT) to cat hair, defined as a wheal diameter ≥ 5 mm compared to the negative control wheal, either at Screening or within 12 months prior to Screening at the research site.

#### Non-allergic participants

To be enrolled into the non-allergic participant group, participants had to have no history of cat-AR and to produce a negative SPT response to all allergens tested in the panel, with the exception of a positive histamine response (Supplementary Data 4), either at Screening or within 12 months prior to Screening at the research site.

### Study design

**Fig. 1:**
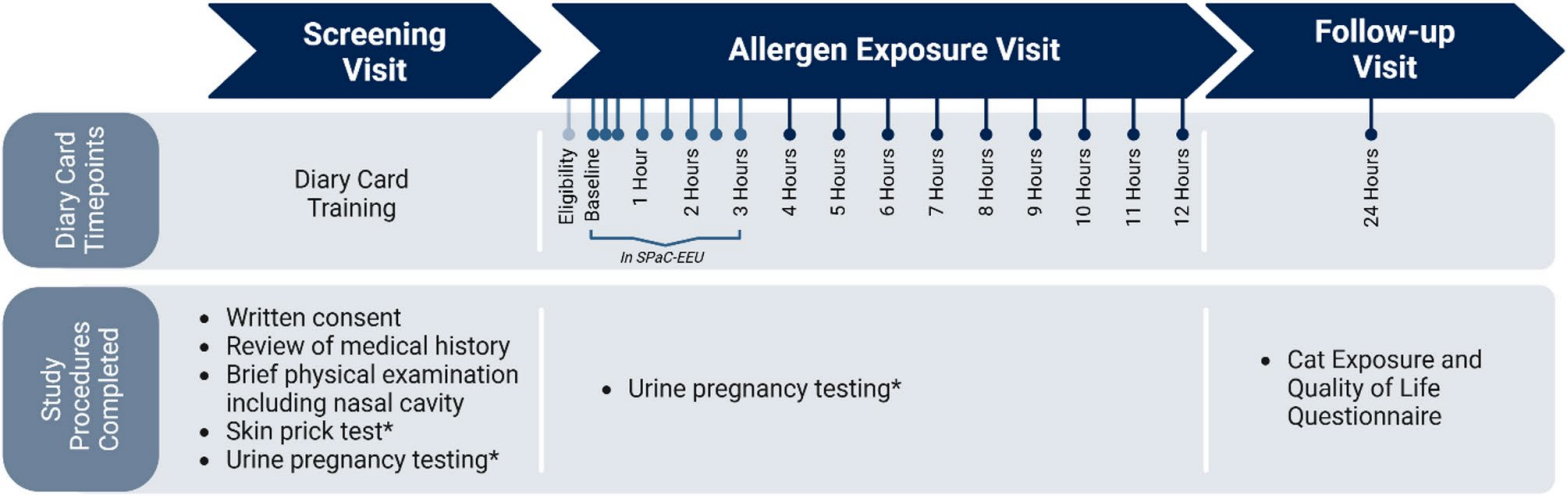
Overview of study design. This study consisted of 3 visits: Screening, Allergen Exposure, and Follow-up. Recruited participants attended Screening to determine eligibility through a review of medical history, a physical exam including of the nasal cavity, and if required, skin prick testing and a urine pregnancy test. Successful participants were invited back to the Allergen Exposure visit, where they were exposed to cat dander for 3 h in the SPaC-EEU. A diary card was collected to assess eligibility. Following a baseline collection, participants recorded symptom scores throughout and after exposure at 18 timepoints. Participants returned to the research site the following day for a 24-hour Follow-up visit. * If required

This study was completed outside of the local pollen seasons and consisted of three visits: Screening, Allergen Exposure, and Follow-up (Fig. 1).

#### Screening visit

After providing written consent, participants had a full medical history review and underwent a brief physical examination, including a detailed nasal examination, with the study physician. Self-reported demographics information was captured. Skin testing was performed for participants without a recent (completed at the research site within 12 months year of the visit) skin test on file. People of childbearing potential underwent a urine pregnancy test to rule out pregnancy. Participants were trained to collect symptom scores and Peak Nasal Inspiratory Flow (PNIF).

#### Allergen exposure visit

Eligible participants were subsequently invited to return to the research site for a 3-hour cat dander exposure session in the SPaC-EEU. Due to space limitations in the facility, participants were divided up to attend one of two exposure sessions (Session 1 and Session 2). Both exposure sessions occurred in the morning, one week apart, and outside of any local pollen seasons. Prior to allergen exposure, participants recorded baseline symptom scores and PNIF and, those of childbearing potential completed urine pregnancy testing.

Following the onset of cat dander exposure in the SPaC-EEU, participants recorded symptom scores and PNIF at 15 min, 30 min, then every half-hour throughout the exposure, and then on an hourly basis post-exposure up to 12 h. Participants remained in the SPaC-EEU for the duration of the 3-hour exposure and were permitted to leave for brief bathroom breaks if needed. Following the completion of exposure and a brief check by the study physician, participants were permitted to leave.

#### Follow-up visit

Participants returned to the research site for a 24-hour follow-up visit. Symptom scores and PNIF were collected. Allergic participants completed a short cat exposure and Quality of Life questionnaire (unvalidated).

Allergic participants were subsequently contacted by phone and/or email to capture details regarding whether or not glasses or contacts were worn during cat allergen exposure in the SPaC-EEU.

### Study procedures

#### Symptom scoring & peak nasal inspiratory flow

Participants were asked to assess a set of 12 symptoms, each on a scale from 0 to 3, increasing in severity. Symptoms were compiled as Total Nasal Symptom Score (TNSS), Total Ocular Symptom Score (TOSS), Total Rhinoconjunctivitis Symptom Score (TRSS), and Safety Scores (Table 1).

**Table 1:** Overview of symptoms assessed

**TRSS**	**TNSS**	• Sneezing • Runny nose/post-nasal drip • Nasal congestion/stuffiness • Itchy nose
	**TOSS**	• Itchy/gritty eyes • Watery/tearing eyes • Red/burning eyes
		• Ear/palate/throat Itching
**Safety Scores**		• Coughing • Wheezing • Chest tightness • Shortness of breath

Peak nasal inspiratory flow (PNIF) was measured using the In-Check PNIF meter (Clement Clark International Ltd, Essex, UK) as per the manufacturer’s instructions. At each assessment timepoint, participants recorded three successful measurements, and the highest measurement was used for analysis.

Both symptom scores and PNIF were recorded on paper diary cards. Participants were trained at the Screening visit on diary card completion and how to correctly use the PNIF device. All diary cards were visually checked for accuracy and completeness during the Allergen Exposure visit. When participants left the research site following the Allergen Exposure visit, they were provided with sufficient diary cards and their personal PNIF meter to complete their remaining assessments at home and were instructed to bring the diary cards back to the site for the Follow-up visit. All data from the paper diary cards and questionnaire responses were manually entered into password-protected Microsoft Excel spreadsheets saved to a secure server. They underwent a 100% quality assurance process. After this, cells were locked to prevent editing. A Quality of Life (QoL) score (out of 12) was generated through the summation of question responses (Table 2), with a higher score suggesting greater interference of cat allergies.

**Table 2:** Quality of Life questions and response options

Questions	Response Options
Do you feel that your cat allergy interferes with your general happiness?	No, never = 0 Yes, sometimes = 1 Yes, always = 2
Do you feel that your cat allergy interferes with your quality of sleep?	No, never = 0 Yes, sometimes = 1 Yes, always = 2
Do you feel that your cat allergy interferes with your productivity?	No, never = 0 Yes, sometimes = 1 Yes, always = 2
Do you feel that your cat allergy interferes with your daily activities at home?	No, never = 0 Yes, sometimes = 1 Yes, always = 2
Do you feel that your cat allergy interferes with your regular daily activities at work/school?	No, never = 0 Yes, sometimes = 1 Yes, always = 2
Do you feel that your cat allergy interferes with your social activities?	No, never = 0 Yes, sometimes = 1 Yes, always = 2

#### EEU methodology

**Fig. 2:**
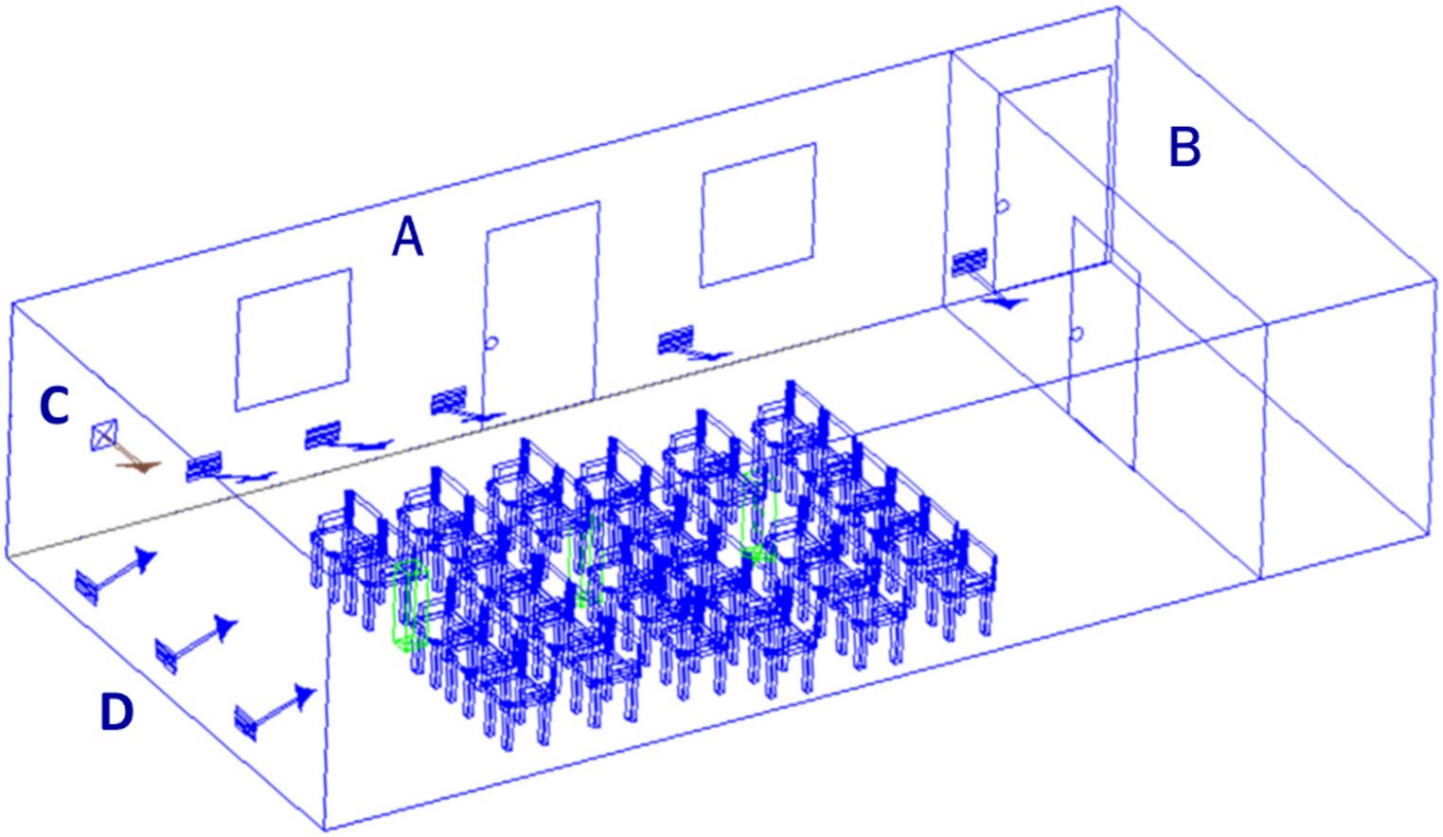
Rendering of the SPaC-EEU. The SPaC-EEU consists of a central room (A), which can seat up to 30 participants, and an anteroom (B), to maintain the appropriate pressure. The influx of allergen is regulated through the feeder (C) located near the front. Perimeter ports (D) ensure dispersed allergen remain active throughout the room. Sampler locations are identified in green

The SPaC-EEU is a specially designed facility at the Kingston Health Sciences Centre in Kingston, Ontario (Fig. 2). It can seat up to 30 participants at one time in the central room. Allergen is distributed into the room through a feeder and distributed using a unique negative pressure system and laminar flow fans. Three 37 mm (Zefon International, Inc., USA) sampling cassettes, located in the front, middle, and back, collected air samples using above-ceiling mounted GilAir5^®^ pumps (Sensidyne, USA) running at 3.1 L/min for the duration of the 3-hour exposure. An additional cassette (pump running at 3.3 L/min) collected air samples near the exit of the SPaC-EEU, in the lobby of the main EEU. Prior to participants arriving for their Allergen Exposure visits, the SPaC-EEU was assessed for endotoxin concentration, using the specific cat dander (Stallergenes Greer, USA) lot obtained. Detected levels (< 3 EU/m^3^) were far below the American Conference of Governmental Industrial Hygienists (ACGIH) threshold limit value (TLV) of 90 EU/m^3^ [21].

Once collected, filters in the sampling cassettes were removed and transferred into 15 mL polypropylene tubes (Sarstedt, Germany) with sterile forceps containing 2 mL of extraction buffer (Dulbecco’s Phosphate-Buffered Saline (Life Technologies, USA) and 0.05% Tween-20 (MP Biomedicals LLC, USA)) for protein isolation. Tubes were shaken at room temperature for 2 h before transferring the buffer into 2.0 mL Protein LoBind tubes (Eppendorf, Germany) for storage at 2-8^o^C. Assessment of Fel d 1 concentration was completed using Fel d 1-specific ELISA (Indoor Biotechnologies Inc., USA).

A laser particle counter (LPC) located at the middle sampler location was used to monitor Fel d 1 concentrations in real-time [22]. The LPC recorded all particles in the air sized 2.5 μm, 5.0 μm, 10.0 μm, 15.0 μm, 20.0 μm, and 25.0 μm for the duration of the exposure.

### Statistical analysis

For data analyses, GraphPad Prism 10.4.1 (San Diego, CA, USA) was used. Given that natural variation exists in raw PNIF scores, the percent change from baseline (%PNIF) was used to assess nasal patency. Participants with missing or incomplete diary cards were excluded from the analysis. Comparisons of symptom scores between cat-allergic and non-allergic participants over time were evaluated using repeated measures two-way ANOVA with Sidak’s multiple comparisons tests. For the analysis of SPT wheal size between cat-allergic and non-allergic participants and Fel d 1 concentrations between Sessions, Mann-Whitney tests were used. Spearman correlations were used to investigate the relationship between two variables. A Kruskal-Wallis test was used to evaluate the differences in QoL scores.

## Results

### Participant demographics are equivalent in Session 1 and Session 2

**Fig. 3:**
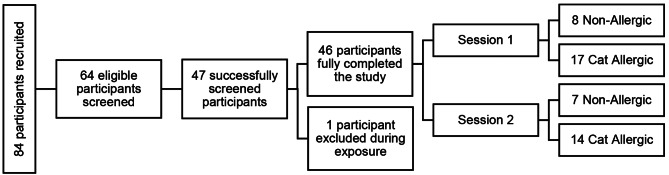
Overview of participant numbers. Of 64 participants, 47 were successfully screened to participate in this study. With one participant excluded during the Allergen Exposure visit, 15 non-allergic and 31 cat-allergic participants fully completed the study

Of 84 participants recruited, 46 successfully completed this study (Fig. 3). Reasons for exclusion included cancellations by participants or no-shows (*n* = 17), ineligibility due to SPT results (*n* = 13) and other inclusion/exclusion criteria (*n* = 7). Additionally, one participant was removed from the SPaC-EEU during allergen exposure by the Principal Investigator due to concerns of asthma-like symptoms. Of 46 participants, 31 were cat-allergic, and 15 were non-allergic controls. Session 1 consisted of 25 participants (17 cat-allergic, 8 non-allergic), whereas Session 2 had 21 participants (14 cat-allergic, 7 non-allergic). The majority of participants were middle-aged (mean age: 42 years old), white, and biologically female, according to their sex assigned at birth (Table 3).

**Table 3:** Demographic details for participants

	Session 1		Session 2		Combined	
	Non-Allergic *n* = 8	Cat Allergic *n* = 17	Non-Allergic *n* = 7	Cat Allergic *n* = 14	Non-Allergic *n* = 15	Cat Allergic *n* = 31
**Biological Sex Assigned at Birth** Male Female	4 4	7 10	1 6	6 8	5 10	13 18
**Mean Age** (SD)	44.75 (12.68)	43.18 (11.93)	38.00 (20.38)	40.71 (12.05)	41.60 (16.45)	42.06 (11.85)
**Race** Black East Asian Indigenous Middle Eastern South Asian Southeast Asian White Other Mixed Race	- - - - - - 8 - -	- - - - - - 15 - 2	- - - - - - 6 - 1	- 1 - - 1 - 12 - -	- - - - - - 14 - 1	- 1 - - 1 - 27 - **2**
**Mean Cat Wheal Size** **(SD)**	0.00 (0.00)	5.91 (1.11)	0.00 (0.00)	6.36 (1.94)	0.00 (0.00)	6.11 (1.53)

### Allergic participants had significantly bigger wheals to cat hair than non-allergic controls

**Fig. 4:**
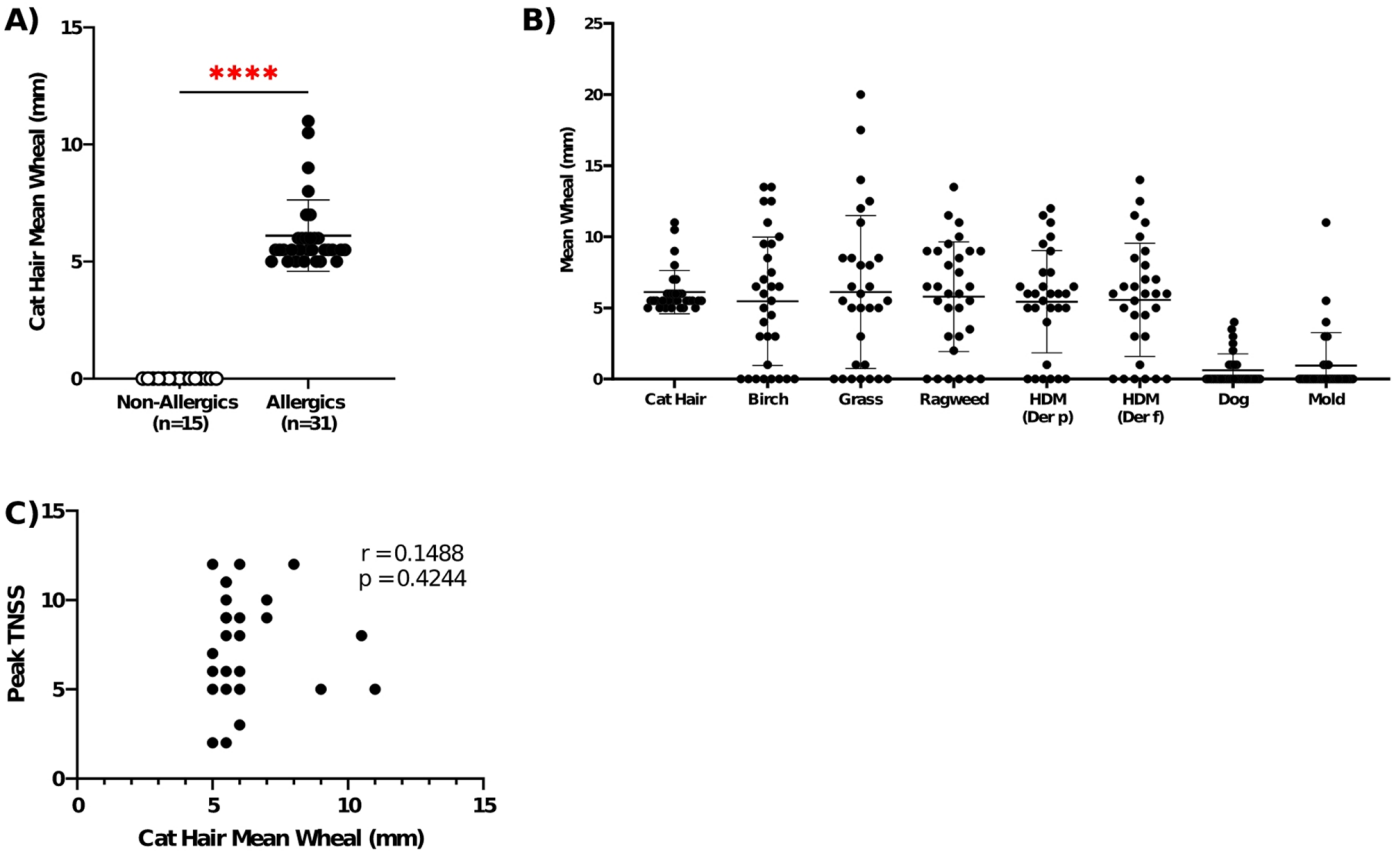
Mean wheals of allergic participants confirm sensitization to cat allergen. **A**) Allergic participants had significantly higher average wheal diameters to cat hair than non-allergic participants. Mann-Whitney test; **** *p* < 0.0001 **B**) The majority of cat-allergic participants (*n* = 29) were polysensitized to other seasonal and perennial allergens. **C**) Mean wheal size for cat hair was not correlated with peak Total Nasal Symptom Scores. Spearman correlation; *p* < 0.05. Error bars are standard error of the mean (SEM)

Cat-allergic participants had significantly greater (*p* < 0.0001) mean wheals to cat hair than non-allergic participants (Fig. 4A).

All except 2 participants were polysensitized to at least one other allergen tested on the SPT panel (Fig. 4B). Of 31 cat-allergic participants, 9 (29%) were co-sensitized to dog allergen and 24 (77%) to HDM. There was no correlation between cat mean wheal sizes and peak TNSS (Fig. 4C).

### Higher concentrations of Fel d 1 distributed in Session 2 than Session 1

**Fig. 5:**
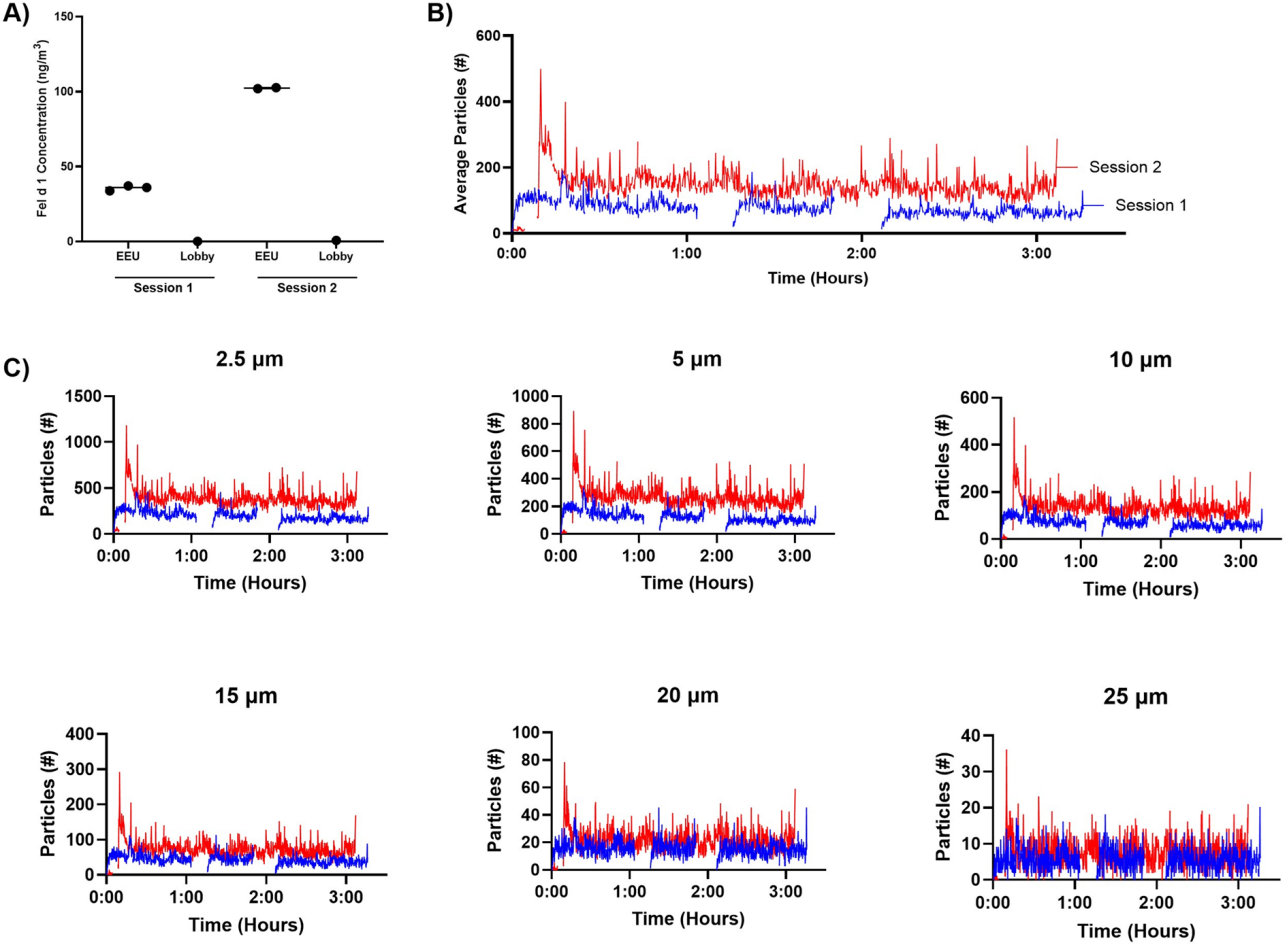
Higher Fel d 1 concentrations and total particle counts were measured during Session 2 than 1. **A**) Mean Fel d 1 concentrations measured from air samples were 35.7 ng/m^3^ and 102.3 ng/m^3^ in Sessions 1 and 2, respectively. In comparison, lobby samples collected from outside the SPaC-EEU were 0.25 ng/m^3^ and 0.81 ng/m^3^. Error bars are SEM. **B**) Particles sized 2.5 μm, 5 μm, 10 μm, 15 μm, 20 μm, and 25 μm were averaged to present average particle counts over time. Session 2 (red) had higher average particles throughout the entire exposure period than Session 1 (blue). **C**) Particle counts in Sessions 1 and 2 plotted by individual particle sizes show higher levels of each in Session 2 than 1. Particles greater than 20 μm in size show stability between Sessions 1 and 2

Mean (SD) Fel d 1 concentrations in Sessions 1 and 2 were 35.7 (1.7) ng/m^3^ and 102.3 (0.5) ng/m^3^, respectively (Fig. 5A). One cassette in the SPaC-EEU did not sample for the entire duration of exposure and was excluded from analysis of Session 2. Air samples in the lobby outside of the SPaC-EEU had 0.25 ng/m^3^ and 0.81 ng/m^3^ of Fel d 1 in Sessions 1 and 2, respectively. Averaging the various sizes of particles, mean counts over time were 76.72 and 146.3 particles for Session 1 and 2, respectively (Fig. 5B). Particles 20 μm and greater showed the most stability between Sessions (Fig. 5C).

### Allergic participants had distinct symptom responses to cat allergen exposure

**Fig. 6:**
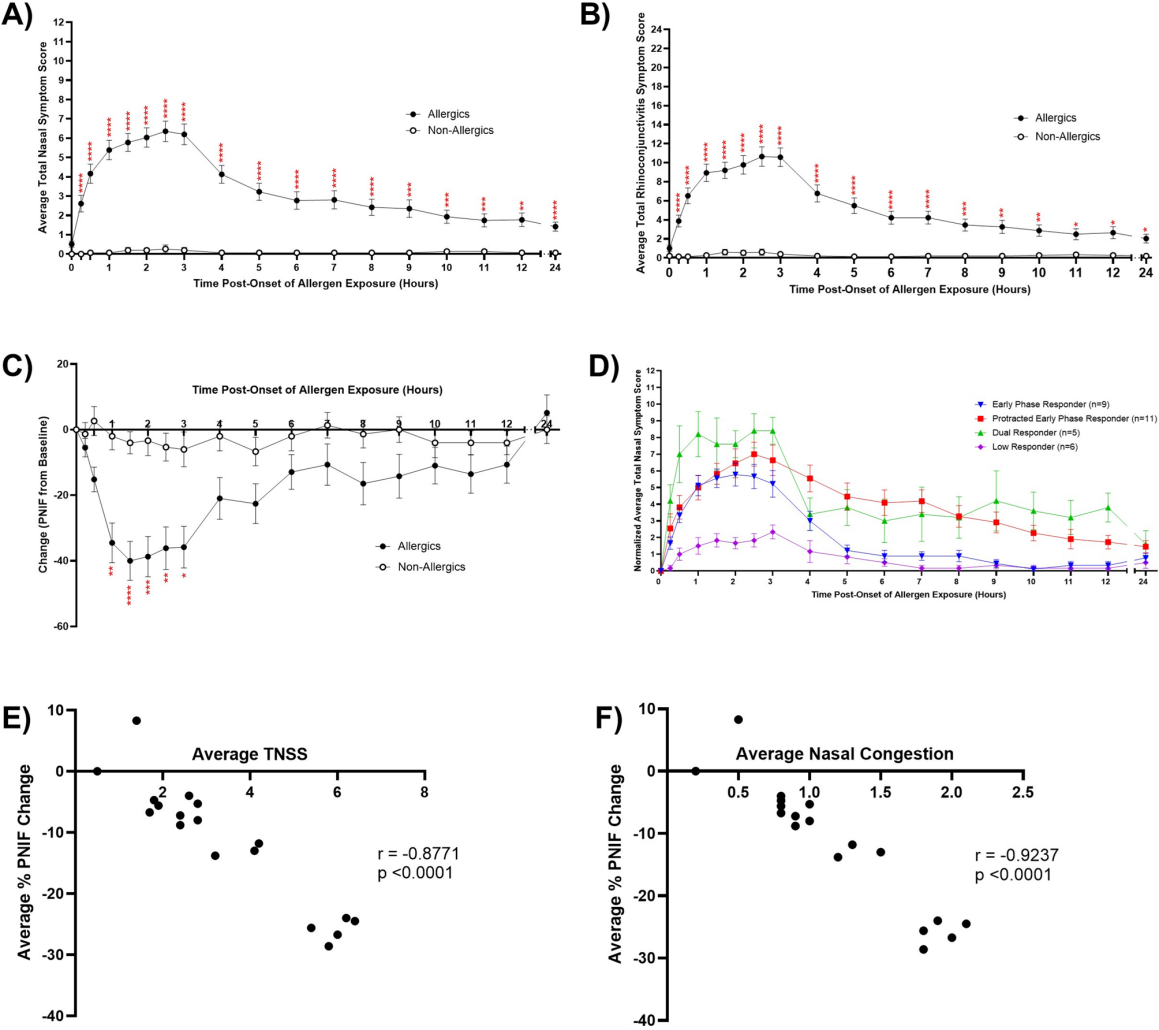
Cat-allergic participants have distinct symptom profiles compared to non-allergic participants. Allergic participants had significantly elevated average A) Total Nasal Symptom Scores (TNSS) and B) Total Rhinoconjunctivitis Symptom Scores in comparison to non-allergic controls at all timepoints, as early as 15 min into the exposure. Repeated measures two-way ANOVA with Sidak’s multiple comparisons test. * p < 0.05, ** p < 0.01, *** p < 0.001, **** p < 0.0001 C) Peak Nasal Inspiratory Flow as a percent change from baseline (%PNIF) was significantly reduced for allergic participants from 1 to 3 hours following the onset of allergen exposure. Repeated measures two-way ANOVA with Sidak’s multiple comparisons test. * p < 0.05, ** p < 0.01, *** p < 0.001, **** p < 0.0001 D) Allergic participants stratified by their late phase response in TNSS were categorized as early phase responders (n = 9), protracted early phase responders (n = 11), dual responders (n = 5), and low responders (n = 6). Average %PNIF was significantly correlated with E) average TNSS (r=-0.8771) and F) average nasal congestion (r=-0.9237). Spearman correlation. p < 0.0001. Error bars are SEM

With no significant differences in TNSS and Safety Scores for matching participants in Sessions 1 and 2, subsequent results were pooled (Figure S1, Fig. 6).

Compared to non-allergic participants, cat-allergic participants experienced significantly elevated (*p* < 0.05) TNSS (Fig. 6A) and TRSS (Fig. 6B) at all timepoints post-exposure (Fig. 6A). %PNIF was significantly decreased (*p* < 0.05) for allergic participants compared to non-allergic participants from 1 to 3 hours during exposure in the SPaC-EEU (Fig. 6C).

Allergic participants were categorized by late-phase TNSS trends into phenotypes (Fig. 6D) [23]. Early phase responders (*n* = 9) did not experience a late phase reaction, protracted early phase responders (*n* = 11) showed continued elevated TNSS hours post-exposure, and dual responders (*n* = 5) experienced a secondary late phase peak in symptoms. Low responders (*n* = 6) did not experience a TNSS greater than 4 at any timepoint.

Significant negative correlations were observed between %PNIF with average TNSS (*r* = -0.8771, *p* < 0.0001; Fig. 6E) and average nasal congestion (*r* = -0.9237, *p* < 00001; Fig. 6F).

**Fig. 7:**
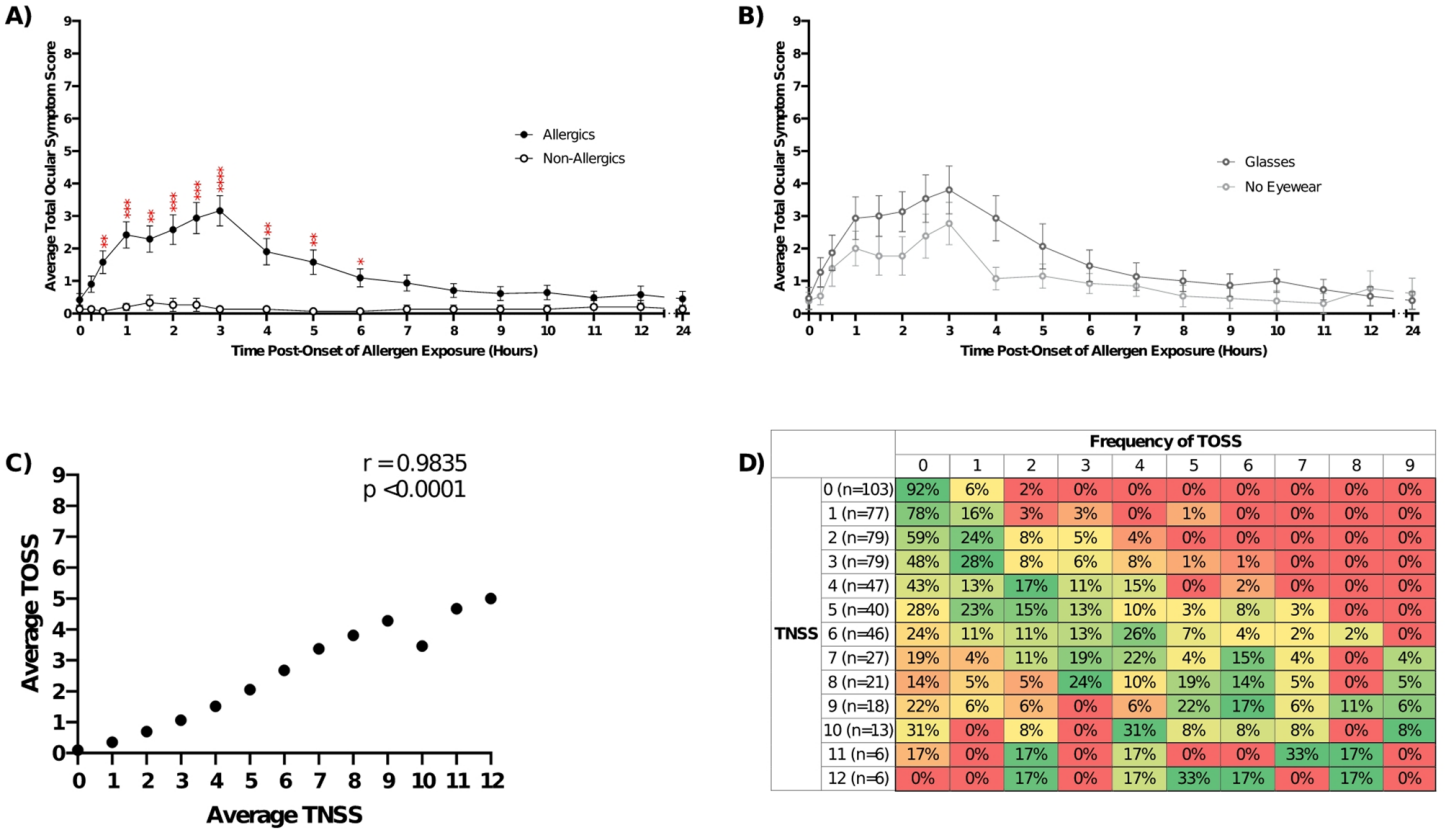
Exposure to cat dander in the SPaC-EEU promoted ocular symptoms in allergic participants. **A**) Allergic participants had significantly increased Total Ocular Symptom Scores (TOSS) from 30 min to 6 h compared to non-allergic controls. Repeated measures two-way ANOVA with Sidak’s multiple comparisons test. * *p* < 0.05, ** *p* < 0.01, *** *p* < 0.001, **** *p* < 0.0001. **B**) The use of eyewear during cat dander exposure had no significant impact on the average TOSS of allergic participants. **C**) Average nasal and ocular symptoms were significantly positively (*r* = 0.9835, *p* < 0.0001) correlated for allergic participants. Spearman correlation. **D**) Ocular symptoms rarely occurred without nasal symptoms, presenting with higher nasal symptom scores. Error bars are SEM

Ocular symptoms, as TOSS, were significantly elevated (*p* < 0.05) for allergic participants compared to non-allergic participants from 30 min into exposure until 6 h post-onset of exposure (Fig. 7A). There was no significant difference in TOSS at any timepoint between allergic participants who wore eyewear during the exposure visit (*n* = 15; all wore glasses) and those who did not (*n* = 13) (Fig. 7B). Ocular symptoms were strongly associated with nasal symptoms (Fig. 7C), presenting with higher nasal symptoms and rarely on their own (Fig. 7D).

There were no significant differences in TNSS at any timepoint when allergic participants were stratified by biological sex assigned at birth, asthma status, drug allergy status, food allergy status, usage of tobacco/e-cigarettes/marijuana, cat ownership, and use of medication for allergies (Figure S2).

### Allergic participants living with a cat reported greater medication usage and interference of sleep

The questionnaire was completed by all cat-allergic participants (*n* = 31). Approximately 52% of participants reported that they grew up being exposed to a cat on a monthly or daily basis.

**Fig. 8:**
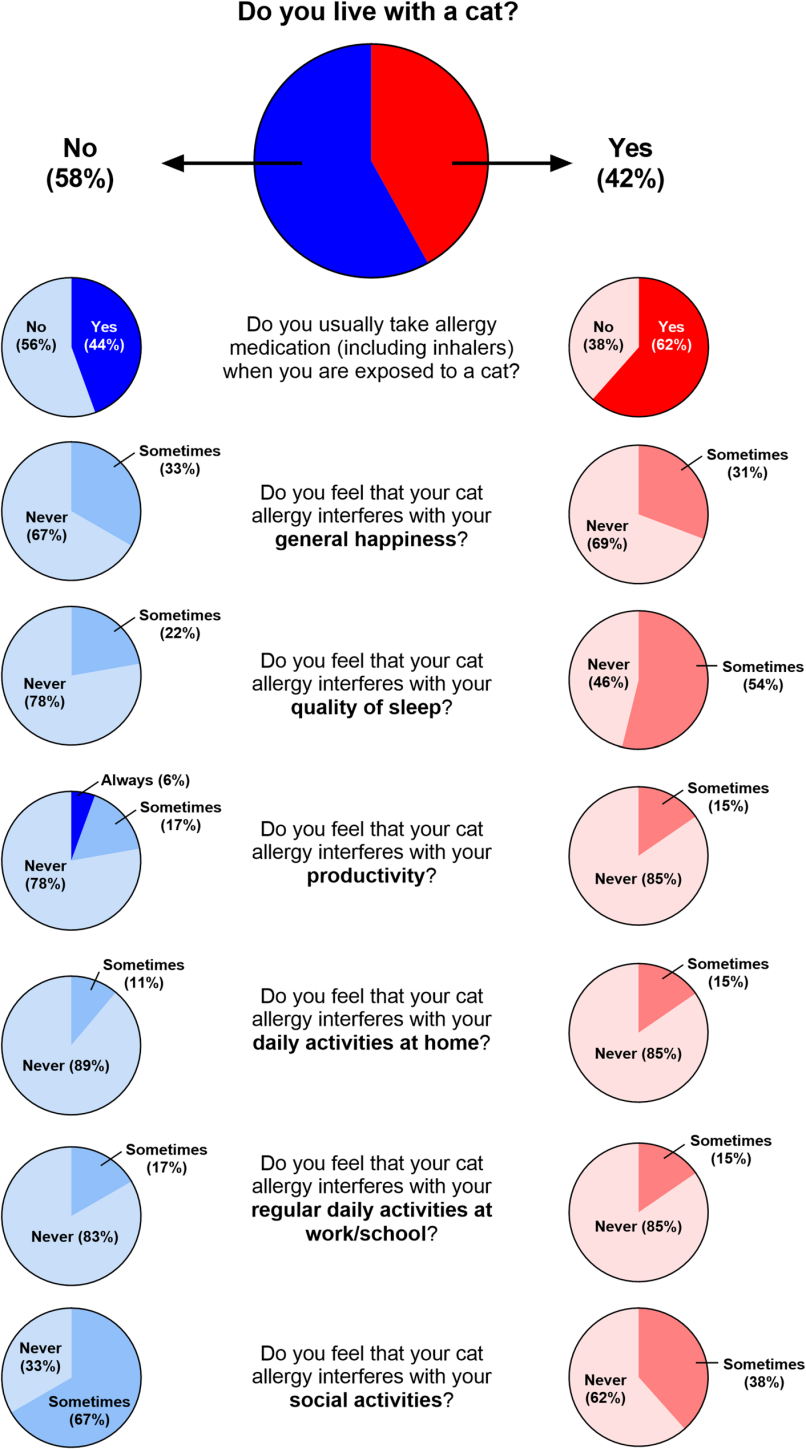
Cat allergic participants’ questionnaire responses. Of our cohort of 31 cat-allergic participants, only 52% reported taking medication upon exposure to cats (**A**). Social activities were reported to be most impacted by their cat allergies (**B**). Approximately 42% of our cohort lived with a cat (**C**)

In our cohort, 13 allergic participants (42%) reported living with a cat, with 62% (*n* = 8) of them reporting that they use medication for their allergies in comparison to 44% (*n* = 8) of participants who did not live with a cat. (Fig. 8). Of participants who use medication for their allergies (*n* = 16), all participants who live with a cat (*n* = 8) reported using medication at least once a week, whereas 7 of the 8 participants who do not live with a cat reported using medication only a few times a month or less.

Regardless of cat ownership, most participants responded that their cat allergy never interfered with their general happiness (67–69%), daily activities at home (85–89%), nor regular daily activities at work/school (83–85%). Allergic participants not living with a cat reported greater interference to their productivity (23% vs. 15%) and social activities (67% vs. 38%) due to their cat allergies than those living with a cat. In contrast, participants living with a cat reported greater interference to sleep quality (54% vs. 22%). Of 7 allergic participants who reported impacted quality of sleep, 5 (70%) allow their cat to sleep in their bed with them. In comparison, only 3 of 6 participants who did not report sleep disturbances (50%) allow their cat to sleep in their bed with them. Participants who did not live with a cat still reported somewhat frequent exposure to cats > 6 months old, whether every few months (39%), once a month (22%), or once a week (17%).

**Fig. 9:**
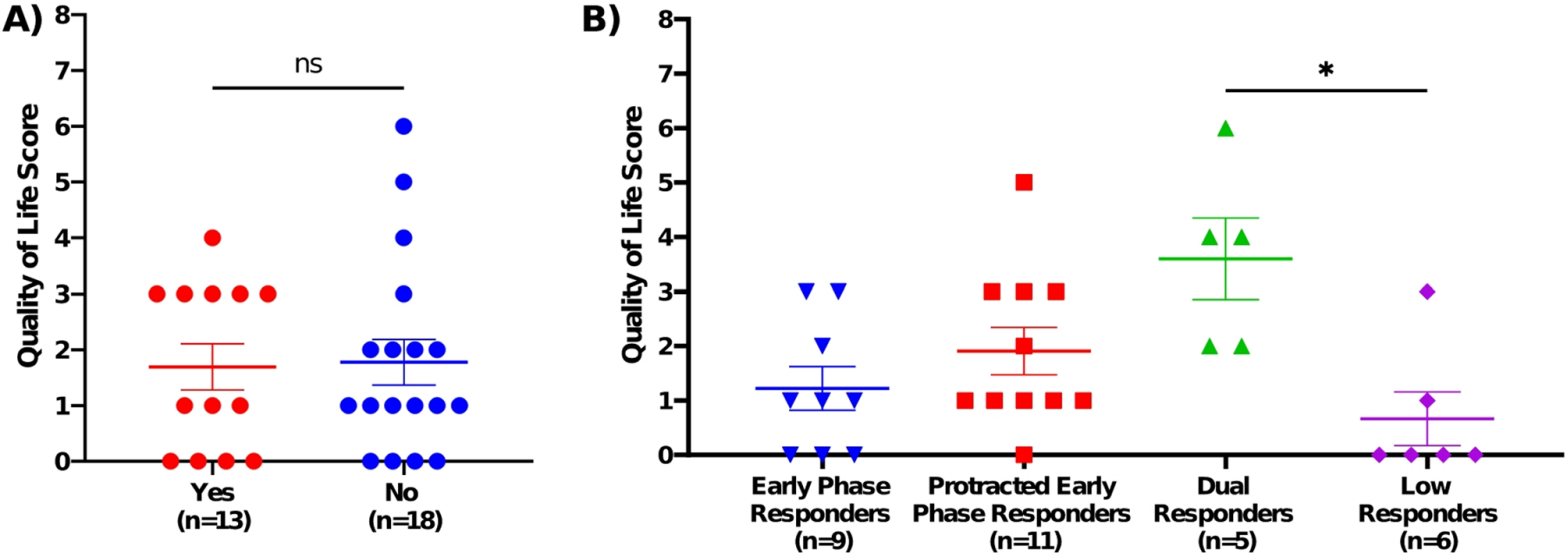
Differential impact of cat allergies through Quality of Life scores. (**A**) No significant differences in QoL were observed between cat allergic participants who lived with or without a cat. Mann-Whitney test; ns *p* > 0.05. (**B**) Dual responders had significantly higher mean QoL scores than low responders. Kruskal-Wallis test; * *p* < 0.05. Error bars are SEM

There was no significant difference in QoL scores when cat allergic participants were stratified by living with or without a cat (Fig. 9A). Interestingly, QoL scores showed meaningful stratification in AR phenotypes (Fig. 9B). Dual responders had the highest mean score (3.6), followed by protracted early phase responders (1.9), early phase responders (1.2), and finally, low responders (0.7). There was a significant different in QoL score between dual responders and low responders (*p* < 0.05).

## Discussion

In this clinical validation study, cat-allergic participants and non-allergic controls were exposed to cat dander in one of two Sessions in the SPaC-EEU with a target of 70 ng/m^3^. Concentrations of the cat allergen Fel d 1 were assessed in air samples collected from sampling cassettes during the 3-hour exposure periods and processed using a Fel d 1-specific ELISA. Session 1 had a mean concentration of 35.7 ng/m^3^ whereas Session 2 had a mean concentration of 102.3 ng/m^3^. This variability is expected with the introduction of human participants into the space. Movement, breathing, sneezing, and the presence of clothing and hair are all factors that can have an impact on airflow and allergen distribution patterns [24, 25]. Despite the variability, both Sessions were within the range of previously reported concentrations of Fel d 1 distributed in other AECs, including the Environmental Exposure Chamber (10 to 500 ng/m^3^) [17] and Naturalistic Exposure Chamber (15.4 to 167.5 ng/m^3^) [18]. Our reported concentrations are also reflective of airborne concentrations of Fel d 1 in the real-world environment. Various studies have investigated Fel d 1 in air samples of homes with cats, with a range of results: 2–20 ng/m^3^ [3], 0.4–22.3 ng/m^3^ [26], 2-468.5 ng/m^3^ [27] and 1.8 to 578 ng/m^3^ [5]. Both the lower and higher ranges of these results are captured by our two exposure Sessions.

The presence of cat allergens is not limited to spaces with cats, however. Cat allergens of detectable concentrations were found in 94% of office buildings assessed by Macher et al. (median Fel d: 0.3 µg/g) despite the seldom presence of cats [28]. In public spaces, Fel d 1 concentrations were 4.5–58 µg/g in upholstered chairs and 0.09–0.22 ng/m^3^ in air samples [29]. The prevalent nature of cat allergens in spaces without cats highlights the ubiquitous nature of the allergen. The negative pressure setup of the SPaC-EEU prevents the dissemination of allergen outside of facility and this was confirmed through air samples collected from the lobby of the EEU, outside of the SPaC-EEU. Fel d 1 concentrations in the lobby of both Sessions were less than 1 ng/m^3^, which is at least 35 times less than distributed concentrations in the SPaC-EEU. This is noteworthy as it confirms the proper functioning of the SPaC-EEU in preventing the distribution of cat allergen beyond the facility into the rest of the hospital. The use of the LPC in the facility enabled real-time monitoring of Fel d 1 concentrations [20]. By being able to simulate a real-world exposure to cat allergen in a controlled manner in the SPaC-EEU, we are able to control extraneous variables while ensuring participant safety.

Despite Session 2 having a higher Fel d 1 concentration and total particle counts than Session 1, allergic participants responded similarly in both Sessions, with no significant differences in TNSS at any timepoint. Interestingly, this is different from our previous findings with HDM, another perennial allergen, whereby allergic participants exposed to the higher HDM target in the SPaC-EEU experienced a significantly higher peak in mean TNSS (*p* < 0.05) compared to those exposed to a modest target [30]. As our goal in this clinical validation is to report symptomatic outcomes from cat allergen exposure in the SPaC-EEU and not to validate specific concentrations, the lack of significant differences between cat-allergic and non-allergic participants in the respective Sessions informed our decision to pool the data. Pooling both Sessions together, cat-allergic participants experienced significantly elevated nasal symptoms and decreased nasal inspiratory flow capacity compared to non-allergic controls. Strong, significant correlations between %PNIF, an objective measure of nasal patency, with subjective symptom scores (TNSS and nasal congestion) confirm accurate reporting of symptoms by participants.

Nasal symptoms reported by allergic participants remained significantly higher even at 24 h compared to controls. This is different from other exposures in the EEU: TNSS of allergic participants was significantly different from non-allergics until 6.5 h post-exposure with birch pollen [31] and up to 5 h with HDM [19]. This is consistent with findings from Murray et al. who reported that persistent nasal symptoms were more common with cat allergies [32]. It is also possible that participants were exposed to other perennial allergens upon leaving the research facility after the Allergen Exposure visit. We did not find significant differences in TNSS between allergic participants who did and did not live with a cat, nor with further comorbid sub-classifications explored, including drug and food allergies. Reported drug allergies included morphine, penicillin, and sulphonamides. Food allergies included nuts, seafood, and pollen food allergy syndrome/oral allergy syndrome.

Allergic participants also experienced significantly elevated TOSS from 30 min to 6 h. Ocular symptoms developed later than nasal symptoms and resolved quickly. In our cohort, approximately 50% allergic participants used eyewear, all glasses. We observed that use of glasses during exposure had no impact on ocular symptom development. Exposure in the SPaC-EEU elicits not only nasal but also ocular symptoms, allowing for a comprehensive study of AR.

As one of the inclusionary criteria for this study, non-allergic participants were required to have a negative SPT for all allergens tested, hence being true controls. This allows us to rule out any hypersensitivity or non-specific source of inflammation with cat dander exposure in the SPaC-EEU. Allergic participants were required to have a positive SPT to cat hair. As a result, it is no surprise that allergic participants have significantly higher cat wheal size than non-allergic controls. Mean wheal size for cat hair was not correlated with peak TNSS, which is consistent with the literature that SPT is a poor predictor of symptom severity [33]. Serological IgE data would be insightful to complement SPT findings.

Unique to other inhaled aeroallergies, pet allergies, specifically cat given its greater prevalence than dog [32], carries a psychosocial burden for allergic participants. Cats are a common household pet in Canada, with around 37% of households owning at least one cat, the majority of which are indoor pets [34]. In a review of the literature by Sparkes, allergies were cited as the second most common reason for cat relinquishment (19%); in comparison, less than 10% of dog relinquishment was due to allergies [35]. In our cohort, 42% of participants lived with at least one cat. Those who live with a cat report greater use of allergy medication (62% vs. 44%) and greater interference with sleep quality (54% vs. 22%) than those who do not live with a cat. Interestingly, allergic participants who did not live with a cat reported greater interference to their productivity (23% vs. 15%) and social activities (67% vs. 38%) due to their cat allergies than those living with a cat.

While overall QoL scores were not significantly different between those who lived with or without a cat, they provided meaningful insight into the AR phenotypes. Dual responders, who experienced a second peak in symptoms at least 6 h after the onset of allergen exposure, were reported to have the highest mean QoL scores, followed by protracted early phase responders, who experienced persistently elevated symptom scores that do not return to baseline by 12 h. Early phase responders, whose symptoms return to baseline shortly after the termination of allergen exposure, had lower mean QoL scores than the first two groups, followed by low responders, who did not experience TNSS greater than 4 at any timepoint. While our questionnaire is currently unvalidated, these findings provide confirmation of its clinical merit.

Approximately half (*n* = 16) of all allergic participants reported that they were frequently exposed to cats growing up on a monthly or daily basis. While some studies have shown early life cat ownership to be associated with reduced risk of wheezing [36]lower skin prick test positivity [37]and sensitization [38, 39]there is conflicting evidence on whether childhood pet exposure is protective or a risk factor for the development of allergic disease [40, 41]. In our cohort, we also observed that approximately 39% of participants who did not live with a cat were still exposed to a cat about once per month or per week. All cats, whether exposure was through living with a cat or not, were reported to be greater than 6 months of age. Age of a cat may change allergen production levels, specifically that older cats may produce less Fel d 1 [42], though findings are inconclusive [43] and other characteristics including fur colour and hair length also do not appear to have an impact on Fel d 1 production [44, 45]. Notably, male cats have been found to have higher allergen levels than female cats, which decreases after neutering, suggesting a hormonal basis for Fel d 1 production [44, 46–48]. The biological purpose of Fel d 1 for cats remains theorized, including providing mucosal protection [2]being a chemical mediator [49]or more recently, having variable functions among different species of cats [50]. However, its role in human health and disease is unequivocal. Our ability to use the SPaC-EEU to clinically model cat-AR is fundamental in furthering our understanding of the disease.

## Conclusions

The SPaC-EEU has been successfully clinically validated for cat dander exposure. Cat-allergic participants experienced significantly elevated nasal and ocular symptoms compared to non-allergic participants over time. No significant safety concerns were identified, rendering the facility a safe and effective model of cat-AR in humans. Further investigations should explore dose-related responses to cat allergen exposure and consider whether the symptomatic effects of controlled cat dander exposure influence biologic changes in participants.

## Electronic supplementary material

Below is the link to the electronic supplementary material.

Supplementary Material 1

## Data Availability

The data supporting this study are available upon request from the corresponding author.

## Abbreviations

ACGIH: American Conference of Governmental Industrial Hygienists
AR: Allergic Rhinitis
Cat-AR: Cat allergen-induced allergic rhinitis
EEU: Environmental Exposure Unit
ELISA: Enzyme-Linked Immunosorbent Assay
Fel d 1: Felis domesticus allergen 1
HDM: House Dust Mite
LPC: Laser Particle Counter
PNIF: Peak Nasal Inspiratory Flow
QoL: Quality of Life
SPaC-EEU: Specialized Particulate Control Environmental Exposure Unit
SD: Standard Deviation
SEM: Standard Error of the Mean
TLV: Threshold Limit Value
TNSS: Total Nasal Symptom Score
TOSS: Total Ocular Symptom Score
TRSS: Total Rhinoconjunctivitis Symptom Score
%PNIF: Percent change in Peak Nasal Inspiratory Flow from baseline
