# Supplementary material for: Clinical validation of controlled exposure to cat dander in the Specialized Particulate Control Environmental Exposure Unit (SPaC-EEU)
Source: Allergy Asthma Clin Immunol. 2025 Aug 6;21:33. doi: 10.1186/s13223-025-00978-z (PMC12330187; doi:10.1186/s13223-025-00978-z)
Supplement: Supplementary file 1 — Supplementary Material 1 [file 13223_2025_978_MOESM1_ESM.docx]

**SUPPLEMENTARY DATA**

**Supplementary Data 1.** Definition of People of Childbearing Potential

**Supplementary Data 2:** Exclusion Criteria

**Supplementary Data 3:** Medication Washout Periods

**Supplementary Data 4:** Skin Prick Testing

**Figure S1.** Participants responded similarly between Sessions.

**Figure S2.** Allergic participants subcategorizations showed no significant differences in Total Nasal Symptom Scores.

**Supplementary Data 1.** Definition of People of Childbearing Potential

A person is considered to be of childbearing potential if they possess functional female reproductive organs, has experienced menarche, has not undergone successful surgical sterilization (hysterectomy, bilateral tubal ligation or oophorectomy), or is not post-menopausal. A person is considered to be post-menopausal if amenorrheal for 1 or more consecutive years.

People of childbearing potential must use an effective method of birth control during the study. These may include oral, transdermal or injectable contraceptives, intrauterine devices or double-barrier methods (diaphragms, condoms plus spermicides), abstinence or a non-heterosexual lifestyle, or those whose partners are sterile (e.g., vasectomized for at least 60 days).

**Supplementary Data 2:** Exclusion Criteria

Participants presenting with any of the following will not be included in the study:

1. Participant has abnormalities detected on physical examination considered by the investigator to be clinically significant and limiting to the study’s participation.
2. Participant has a history of any disease or disorder that, in the judgement of the investigator, would impact on participant’s safety or the results of the study.
3. Participant has a significant history of alcohol or drug abuse in the judgment of the Principal Investigator or delegate.
4. Participant that is pregnant, lactating, or actively trying to become pregnant.
5. Participant is unable to comply with the washout periods for restricted medications (Refer to **Supplementary Data 3: Medication Washout Periods**).
6. Participant has signs/symptoms of active seasonal allergic rhinitis or is allergic to a seasonal allergen that is present in the outdoor environment during the time of the SPAC-EEU exposure visit and which the Principal Investigator judges would impact the outcome of the study
7. Participant has any structural nasal abnormalities or nasal polyps on examination, a history of frequent nasal bleeding, or nasal surgery within the previous 3 months before screening, as determined by the Principal Investigator to potentially interfere with study outcomes.
8. Participant has experienced an upper or lower respiratory infection within 2 weeks prior to the challenge visit.
9. Participants with asthma requiring the use of a short-acting beta agonist greater than twice a week (unless for viral or exercise induced asthma) or with severe asthma requiring maintenance high dose of inhaled or oral corticosteroids or biologic therapy (Omalizumab/Mepolizumab/Resilizumab).
10. Participant has a history of cat allergen induced asthma, unless well controlled on low dose inhaled corticosteroid or PRN inhaled corticosteroid/long-acting beta-agonist or as per Principal Investigator discretion.
11. Participant is currently receiving cat allergen specific immunotherapy or concluded a course of cat immunotherapy in the last 3 years.
12. Participant has a history of positive test results for to HIV, Tuberculosis (not due to vaccination), Hepatitis B (not due to vaccination) or Hepatitis C.
13. Participant has received an investigational product within the previous 30 days.
14. Participant is unable and/or unlikely to comprehend and/or follow the protocol over the duration of the study.
15. Participant is unwilling to attend study visits or adhere to the study protocol, in the judgement of the investigator.
16. Participant exhibits any signs or symptoms of COVID-19 or has a positive test.
17. TNSS>/= 4 at baseline visit 2 as per PI judgement.*

* If TNSS >/= 4 at baseline, participant may be re-booked if space available.

**Supplementary Data 3:** Medication Washout Periods

Restricted Medications: All medications, including herbal, homeopathic, naturopathic and other dietary supplements, taken by the participant from 30 days prior to Screening through until the end of the study will be documented. The reported medications will be reviewed and evaluated by the Principal Investigator or designee to determine if they affect the participant’s eligibility to participate in the study.

| **Medication** | **Duration of Washout prior to**  **Cat Allergen Exposure Visit** |
| --- | --- |
| Beta-blockers | NOT PERMITTED |
| Decongestants (oral and topical) | 48 hours |
| Short-Acting Antihistamines (e.g. Diphenhydramine) | *3 days |
| Anti-allergic eye drops (ocular antihistamines, decongestants and cromoglycates) | 3 days |
| Long-Acting Antihistamines (eg. fexofenadine, loratadine, cetirizine) – note that H2 antagonists (e.g. Zantac, ranitidine) are not considered antihistamines for the purposes of this study | *5 days |
| Intranasal anticholinergics | 3 days |
| Intranasal corticosteroids (eg. budesonide, fluticasone etc.) | **14 days |
| High dose inhaled corticosteroids (eg fluticasone propionate >500ug or equivalent) | 14 days |
| Mast cell stabilizers | 5 days |
| Leukotriene inhibitors | 14 days |
| Tricyclic Antidepressants and Monoamine oxidase inhibitors | NOT PERMITTED |
| Systemic corticosteroids (oral) | 30 days |
| Injectable corticosteroids | 60 days |
| Biologics | 6 months |
| Tobacco and/or marijuana | 12 hours |

**Supplementary Data 4:** Skin Prick Testing

Skin prick testing was performed to determine the participant’s allergic history, if not on file from the 12 months prior to Screening. This testing was done following our research group’s Standard Operating Procedures, administered using sterile plastic bifurcated Duotip-Test II devices. In addition to a histamine positive control and a glycerin phenol-saline negative control, the test panel included the following allergens: Standardized Cat Hair (Hollister-Stier; 10,000 BAU/mL), Birch (ALK-Abelló; weight per volume (w/v) 1:20), Timothy Grass (ALK-Abelló; 100,000 bioequivalent allergy units (BAU)/mL), Short Ragweed (ALK-Abelló; w/v 1:20), D. pteronyssinus, D. farinae (ALK-Abelló; 10,000 AU/mL), Dog Epithelium (ALK-Abelló; w/v 1:20), and Mold Mix (ALK-Abelló; w/v 1:20).

**Figure S1. Participants responded similarly between Sessions.** No significant differences in average Total Nasal Symptom Scores (A, B) and Safety Scores (C) between matching participants in Session 1 and 2. Repeated measures two-way ANOVA with Sidak’s multiple comparisons test. Error bars are SEM.

**Figure S2. Allergic participants subcategorizations showed no significant differences in Total Nasal Symptom Scores.** No significant differences in allergic participants were observed when stratifying by biological sex assigned at birth (A), self-reported asthma (B), self-reported drug allergies (C), self-reported food allergies (D), smokers of tobacco/e-cigarettes/marijuana (E), the presence of a cat at home (F), and medication usage (G). p>0.05, Repeated measures two-way ANOVA with Sidak’s multiple comparisons test. Error bars are SEM.
